# The Renal Status of Opium Habitués1Read before Philadelphia County Medical Society, by invitation, October 22, 1890.

**Published:** 1891-01

**Authors:** J. B. Mattison

**Affiliations:** Brooklyn; Member American Association for the Cure of Inebriety; of the New York Neurological Society; of the Medical Society of the County of Kings


### THE RENAL STATUS OF OPIUM HABITUÉS.[1]

By J. B. MATTISON, M. D., Brooklyn.

Member American Association for the Cure of Inebriety; of the New York Neurological Society; of the Medical Society of the County of Kings.

This paper enters a field which, so far as I am aware, has not been largely worked at home, and is preliminary to one that will include, it is hoped, the opinions of other leading pathologists here and abroad.

Its present scope is, mainly, the views of American observers whose high repute entitles them to large respect. It will present opinions pro and con; it will offer such clinical, chemical, and microscopical evidence as has passed into history touching the topic under discussion; and it will give details of some pertinent cases that have come within the reader's personal range.

Until recent years it was largely held that while the habitual use of opium gave rise to well-marked, and often grave functional disorder, it did not, like alcohol, cause structural lesion. Of late, however, there has been a growing consensus of opinion that this view is a mistaken one, and that, in a fairly large proportion of cases, its continued taking — especially morphia hypodermically — will induce renal changes ranging from slight hyperemia to profound organic derangement, ending in death.

Reaching the pith of this question, three queries were presented:

Will the habitual use of opium, in any form, cause organic renal disease?

If so, what lesion is most likely?

What is the *rationale?*

Roberts Bartholow: I have made numerous observations on the effects of opium on the urine, but with negative results. The reason for this appears to me to be rational. The action of opium or morphine, used habitually, tends to diminish excretion by lessening the activity of the kidneys. The effect on the kidneys is, therefore, rather soothing and grateful to these organs. In lessening action, the blood-supply of the kidneys is diminished, congestion is prevented, and the organs carry on their functions with less strain.

Francis Delafield: It has not seemed to me that opium is a cause of any kidney disease.

Thomas E. Satterthwaite: In two post-mortems of patients who died of opium poisoning, the kidneys showed no structural

1. Read before Philadelphia County Medical Society, by invitation, October 22, 1890.

change, although in one patient these organs were swollen and congested.

HERMAN M. BIGGS: Of all the autopsies that I have seen in these cases, I have been impressed with the marked chronic congestion of the kidneys. This is frequently, and, I think, generally, associated with a uniform increase in the interstitial connective tissue of the organs. The kidneys are of a very deep, uniform red color, and the consistence is decidedly increased. But I very much doubt whether the habitual use of opium in itself will cause extensive organic disease of the kidneys; I mean such disease as will seriously compromise their functions. I have never seen a case of opium addiction at the autopsy, where extensive disease of the kidneys was found, that I believed was due to the effect of the opium. I do not think the changes I have stated, as a rule, seriously interfere with the performance of the renal functions.

E. H. BARTLEY, Professor of Chemistry and Toxicology, Long Island College Hospital: So far as my observation goes, I have seen no reason to believe that the alkaloid exerts any direct influence to produce organic disease of these organs. It seems to be eliminated easily and rapidly by the kidneys, and without irritation. This is especially the case when given by the stomach, in which case it is eliminated almost as fast as it is absorbed. If the practice of opium or morphine-taking has any influence in producing organic disease of the kidneys, it is rather to be regarded as an accidental or indirect one. While morphine does not seem to possess any irritant action upon the renal structure, it lessens the amount of urine passed, it retards oxidation and the elimination, and, probably, the formation of urea. Urine, so modified, is more irritating than normal, less concentrated urine, and may possibly tend to the formation of the gouty kidney, as interstitial nephritis. This effect is aggravated by the disordered digestion induced by the alkaloid. When morphine is self-administered, by means of the hypodermic syringe, another accidental influence is at work. Without proper care of the syringe as to cleanliness, abscesses occasionally occur at the seat of the injection, and these tend to produce nephritis of septic origin.

JOHN A. MCCORKLE, Professor of Theory and Practice, Long Island College Hospital: I think the habitual use of opium will cause organic renal disease; that the small granular kidney is most likely, and that the reason is a low grade of inflammation induced by the elimination of a foreign substance.

J. H. VAN COTT, JR., Pathologist, Long Island College Hospital: Based upon the supposed physiological action of opium and its alkaloids, I believe that its habitual use must be productive of renal disease. Its direct action on the vaso-motor apparatus must finally result in chronic dilation of such vessels as are controlled by the nervous system and contain non-striated muscular fibres.

Its effect upon the digestive apparatus, and the well-known disturbance of primary and secondary assimilation, can hardly eventuate otherwise than in the most profound disturbance of all the so-called parenchymatous tissues; a disturbance perhaps well expressed by the term faulty retrograde metamorphosis. From any reasonable standpoint the metabolism of cells is bound to be inhibited by the action of these narcotics, partly through vaso-motor disturbance, also through the *direct* effect of the drug on cell activity.

If, on the one hand, conditions of profound nutrition and vascular disturbance are brought about to inhibit the proper metamorphosis of all the tissues in the body, yielding only poorly constituted products for elimination; if, in other words, the true "end-products" are not attained; and if, on the other hand, the metabolic action of the very cells whose business it is to eliminate these "end-products" is inhibited — if the epithelial cells of the renal parenchyma are thus handicapped, it is inconceivable to me that they should not in time become permanently modified both in form and in functional activity.

Theoretically, the renal changes should be all those resulting from chronic renal hyperemia — a peculiar form of diffuse nephritis, with an added parenchymatous degeneration.

Two cases of the opium disease have come under my observation, in both of which grave renal symptoms obtained. One died, and the autopsy revealed widespread chronic lesions of all the organs, the kidneys not excepted; the other is still living, having had in the course of the Summer a uremic attack, which nearly proved fatal.

I cannot dogmatize on the subject until more experience has been gained in the thorough, systematic, and impartial observation of the renal functions in such cases; but my experience, such as it is, all points toward the correctness of my reasoning.

Text-books make little or no mention of this topic. Wilson in an excellent paper — Pepper's *System* — refers to it briefly. Niemeyer tells of interstitial nephritis due to "septic material in the blood." Roberts writes of albuminuria due to disturbed innervation. Bartholow, in *Hypodermic Medication*, gives negative evidence as to albumin and sugar in urine due to morphia, and thinks Levinstein's statements not applicable to cases met in this country. He further says — and this is of no mean moment as pointing to a factor in renal break-down :

> The frequent use of the syringe, often the hasty introduction of the needle, and the use of a rusty and dirty needle, the injection of badly prepared solutions, the repeated injections into certain localities, have a disastrous effect. Large hard nodules form, which slowly suppurate. Extensive sloughing may take place, and septicemia and pyemia sometimes occur with a fatal result. In a large proportion of these morphiamaniacs, suppuration abscesses of considerable size, and ulcers, are produced. I have often seen the arms, the abdomen, the thighs, and legs, a mass of ulcers and of abscesses in various stages of formation.

Dujardin-Beaumetz cites a fatal case of this kind, and Calvas has noted many such with traumatic fever.

The most extensive, interesting, and valuable notes on this point are those of Levinstein, who observed many cases, and made frequent trials on rabbits and dogs, always getting albumin, and usually sugar. The latter he regarded as specially significant, and its relation to morphia-taking, acute or chronic, of high medico-legal importance, as strong presumptive proof of the toxic agent in cases of suspected fatal poisoning from that drug.

In the absence of positive pathological facts, Levinstein thought that albumin might be due to one or all of three causes :

1. The action of morphia on the central organ, which has a relation to albumin.

2. By the concurrence of anomalies in the normal pressure of the blood, causing at last intra-renal disorders.

3. By paralysis of the nerves which surround the arteria renalis. This would have the same effect as knife-cutting of those nerves, after doing which, von Wittich found albumin in the urine.

Levinstein thinks it very probable that varying blood-pressure is the leading factor. This, he suggests, explains the intermittent albuminuria common in these cases, while *continuous* albumin is due to nerve-palsy of the renal artery.

Autopsic opportunities in these cases are not frequent, and the following, under the notice of Dr. Van Cott, and reported in the October Brooklyn *Medical Journal*, is of interest :

> Mrs. B., aged forty years ; semi-invalid for years, during the last four of which she used morphia subcutaneously ; was attacked with sudden illness, having persistent vomiting, some purging, scanty, high-colored, albuminous urine, which progressively increased. Later, clonic spasms of left arm, extending to entire body, like uremic convulsions ; urine almost suppressed ; extremities edematous. Death by coma and convulsions.

*Necropsy.* — Left kidney larger, right smaller than normal; soft; surface studded with countless small punctiform hemorrhages. Around Malpighian corpuscles numberless hemorrhages. Microscopic — fresh, double-knife section: Most of glomeruli are hemorrhagic, extravasated blood being seen around the Malpighian tufts within Bowman's capsule. Cortex and medulla markedly congested. Epithelial cells in many tubes desquamated ; many tubules have hyaline, epithelial, and granular casts ; others, much granular detritus.

Conclusion : Case of microbic infection. Patient, in self-giving of morphia, drove needle through pus in left deltoid, causing systemic infection, and over-strain on kidneys already crippled by opium excess.

Since September 1st, eight cases of the morphine disease have been under the writer's care, in which careful chemical and microscopical tests were made. Of these, seven gave proof of renal changes ranging from chronic hyperemia to structural lesion and death.

Case I.— Physician ; aged thirty-six years ; kindly referred to me by Dr. Bartholow. Four years' morphia-taking — eight grains subcutaneously daily. Examination disclosed such evidence of profound kidney changes that no attempt at treatment of his morphia-using was made. Patient remained a fortnight, when he showed signs of general failure, which ended in death on the eighteenth day, from lung edema, of about twelve hours' duration.

Case II. — Physician ; aged thirty years ; four years' morphia-taking ---thirty grains daily in one dose, hypodermically. Quit treatment prematurely, and was forced to resume the drug ; is again under treatment, and will recover.

Case III. — Layman ; aged thirty years ; four years' morphia-taking --- nine grains subcutaneously ; recovered.

Case IV. — Clergyman ; aged forty-nine years ; twenty-seven years' addiction—twenty-three years opium, four years' morphia, by mouth, six grains daily ; recovered.

Case V. — Female ; aged thirty-four years ; ten years' morphia-taking—eighteen grains daily, hypodermically ; recovered.

CASE VI.—Physician; aged twenty-eight years; four years' morphia-taking—sixteen grains, subcutaneously, daily; unrecovered.

CASE VII.—Layman; aged seventy years; three years' hypodermic taking—four grains daily; recovered.

CASE VIII.—Physician; aged thirty years; one year, irregular, subcutaneous taking—three grains daily; recovered.

Of these eight cases, seven had crippled kidneys, and all used the drug by needle. The twenty-seven years' taker by mouth showed no kidney lesion.

Repeated chemico-microscopical searchings in such cases have disclosed certain changes from normal renal action, and so often, that we think they may be accepted as the rule.

The urine is diminished.

The urea is lessened.

Specific gravity is varied; if changed, usually lowered.

Albumin is present.

Sugar also.

The chlorides are decreased.

Casts,—granular,—hyaline, and waxy,—are present.

Urine sediment holds epithelial cells and granular detritus.

Concluding, our own opinion, in reply to the three-fold query, is:

*First:* Yes.

*Second:* Cirrhotic.

*Third:* A triple factor—vasomotor changes; impaired general nutrition, and inflammatory action due to non-eliminated irritant products.

